# Initial Experience with the Saroa Surgical System in Robot-Assisted Hysterectomy: First Clinical Case Series and Haptic Feedback Assessment

**DOI:** 10.3390/medicina61091716

**Published:** 2025-09-21

**Authors:** Noriko Oshima, Naoyuki Yoshiki, Yusuke Kohri, Maki Takao, Naoyuki Miyasaka

**Affiliations:** Department of Obstetrics and Gynecology, Institute of Science Tokyo, Tokyo 113-8510, Japan; n.yoshiki.crm@tmd.ac.jp (N.Y.); k-yucrm@tmd.ac.jp (Y.K.); t-maki.crm@tmd.ac.jp (M.T.); n.miyasaka.gyne@tmd.ac.jp (N.M.)

**Keywords:** Saroa surgical system, robot-assisted hysterectomy, haptic feedback, minimally invasive surgery

## Abstract

*Background and Objectives*: Laparoscopic surgery has evolved with the integration of robotic systems, offering enhanced precision and ergonomic benefits. However, conventional robotic systems often lack haptic feedback and are associated with high cost. The Saroa surgical system is a compact, pneumatically driven robot that integrates real-time haptic feedback, potentially addressing the limitations associated with conventional robotic systems. This preliminary study reports the first clinical use of the Saroa system in gynecologic surgery, aiming to assess its feasibility, safety, and usability in robot-assisted hysterectomy. *Materials and Methods*: Five patients underwent robot-assisted total laparoscopic hysterectomy using the Saroa surgical system. The clinical outcomes, setup and console times, estimated blood loss, and subjective surgeon evaluation were recorded. *Results*: All surgeries were successfully completed without any intraoperative complications or the need for conversion to conventional surgery. The median setup time was 12 min, the console time was 211 min, and the median blood loss was 80 mL. Surgeons subjectively noted that the system’s real-time haptic feedback substantially improved precision during vaginal cuff tissue manipulation, based on their tactile sensation and real-time force display, thereby reducing the perceived risk of traction-related tissue injuries. *Conclusions*: This study represents the first clinical application of the Saroa surgical system in gynecologic surgery. The findings suggest that the system is feasible and safe for robot-assisted hysterectomy. Despite limitations such as small sample size and the absence of objective force data, the favorable surgeon-reported experience highlights the potential value of haptic feedback in improving surgical performance. These results support further investigation through larger, controlled studies and quantitative performance evaluation.

## 1. Introduction

Minimally invasive surgery is regarded as the gold standard for the treatment of gynecological conditions. Although robot-assisted surgery (RAS) offers enhanced precision and visualization, conventional systems often lack haptic feedback, which may impair delicate tissue handling and increase the risk of inadvertent injury [1]. The Saroa surgical system (Riverfield Inc., Tokyo, Japan), introduced in 2023, represents a next-generation robotic surgical platform specially engineered to overcome these limitations. Unlike previous systems, the Saroa surgical system utilizes a pneumatically actuated mechanism that provides real-time haptic feedback, restoring the surgeon’s ability to perceive the grasping force during manipulation [2]. The compact and lightweight configuration of the system, coupled with its streamlined setup, offers significant logistical and cost-related benefits.

The development of the Saroa surgical system originated from a collaborative research initiative between the former Tokyo Medical and Dental University and the Tokyo Institute of Technology (now Institute of Science Tokyo). However, during the development phase, validation specifically focused on gynecologic procedures had not been conducted. This study represents the first report worldwide on the clinical application of this system in gynecologic surgery.

Our study presents the initial clinical experience with the Saroa surgical system in robot-assisted hysterectomy (RAH) and evaluates its feasibility, safety, and integration within contemporary laparoscopic workflows.

## 2. Materials and Methods

### 2.1. Patient Selection

This study enrolled five consecutive patients who underwent robot-assisted laparoscopic hysterectomy using the Saroa surgical system between October 2023 and December 2024 at the Institute of Science Tokyo Hospital. The inclusion criteria encompassed benign gynecological conditions warranting hysterectomy, including uterine myoma, endometrial hyperplasia, and endometrial polyps. The exclusion criteria included malignancy, pregnancy, and the inability to tolerate pneumoperitoneum. The study was approved by the Institutional Ethics Committee, and written informed consent was obtained from all patients. Clinical data, including patient demographics, operative duration, blood loss, complications, and device performance, were retrospectively collected.

### 2.2. Preoperative Preparation

All patients underwent standard preoperative evaluations, including imaging, laboratory testing, and anesthesia clearance. Additionally, prophylactic antibiotics were administered preoperatively, according to the institutional protocol. Informed discussions with the patients included specific consent regarding the use of the novel surgical system.

### 2.3. Surgical Procedure

**Figure 1:**
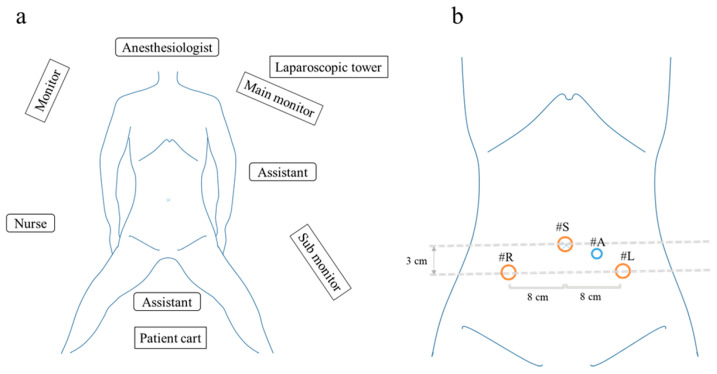
Setup and trocar placement in robotic-assisted hysterectomy using the Saroa system. (**a**) Operating room layout illustrating the compact patient cart positioned between the patient’s legs. (**b**) Trocar configuration displaying camera port at the umbilicus, with instrument ports placed laterally and caudally. R, Right arm; S, Scope arm; L, left arm; A, Assistant port.

A schematic of the surgical setup and port placement is provided in Figure 1.

After induction of general anesthesia, the patients were placed in the lithotomy position with a 15-degree Trendelenburg tilt. The overall layout of the operating room is shown in Figure 1a. To optimize access to the three robotic arms, the patient cart was positioned on the patient’s left side. The first assistant stood on the patient’s left side to perform suction, retraction, and instrument exchange, while the second assistant was positioned between the patient’s legs to operate the uterine manipulator. The scrub nurse was positioned on the opposite side of the first assistant. The anesthesiologist was located at the head of the patient, with a main monitor positioned for shared viewing by the assistants and scrub nurse, and a sub-monitor providing supplementary visual information. The laparoscopic tower was placed near the anesthesiologist to facilitate easy access to equipment. A 12 mm trocar was inserted through the umbilicus, followed by the placement of two 11 mm trocars, each positioned 8 cm lateral and 3 cm caudal to the umbilical port on each side (Figure 1b). A 12 mm trocar was inserted through the umbilicus, followed by the placement of two 11 mm trocars, each positioned 8 cm lateral and 3 cm caudal to the umbilical port on each side. An additional 5 mm port for the auxiliary forceps was inserted at a point midway between the umbilical and left lateral abdominal ports. A 10 mm, 30-degree oblique-view laparoscope (Olympus, Tokyo, Japan) was introduced through the umbilical port. All trocars used were ENDOPATH XCEL™ bladeless trocars (ETHICON, Johnson & Johnson, Cincinnati, OH, USA).

**Figure 2:**
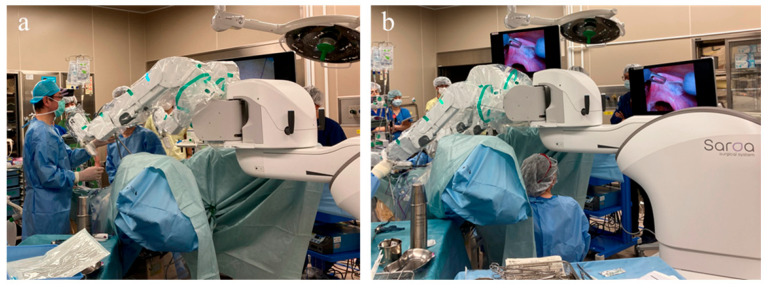
Intraoperative setup of the Saroa surgical system during robot-assisted hysterectomy. (**a**) The robotic arms are positioned over the patient, with the compact patient cart positioned between the legs, allowing unobstructed access for the surgical assistants. (**b**) Adequate space is maintained beneath the robotic arms to accommodate a second assistant, allowing for the use of a vaginal pipe or uterine manipulator when required.

The cart for the Saroa surgical system was subsequently positioned between the legs (Figure 2a), allowing unobstructed access for the surgical assistants. Adequate space was also maintained beneath the robotic arms (Figure 2b) to accommodate a second assistant, enabling the use of a vaginal pipe or uterine manipulator when required.

**Figure 3:**
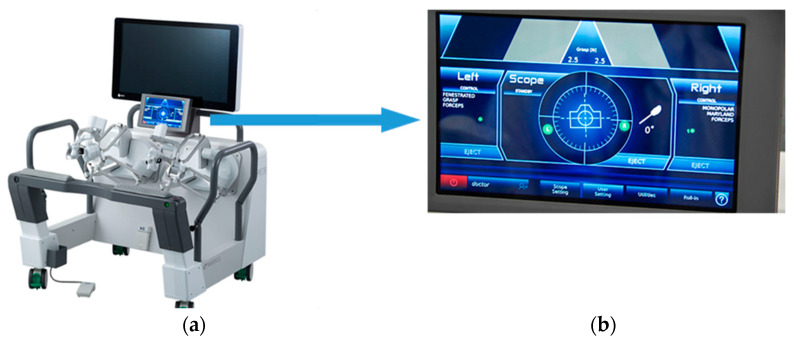
Surgeon console and navigation monitor of the Saroa surgical system. (**a**) The surgeon operates from an open console wearing three-dimensional glasses, utilizing hand controllers that provide real-time haptic feedback to facilitate precise instrument manipulation. (**b**) A navigation monitor is used during the procedure. The monitor provides real-time visualization of endoscopic images and system status, allowing coordination between the console surgeon and the surgical assistant.

Due to its compact size, the system can be rolled in with ease, providing unobstructed access to the surgical field. The instruments were mounted on the three robotic arms of the Saroa surgical system and inserted through the ports without docking. The surgeon performed the procedure at an open console, wearing three-dimensional (3D) glasses and operating hand controllers equipped with real-time haptic feedback (Figure 3).

**Figure 4:**
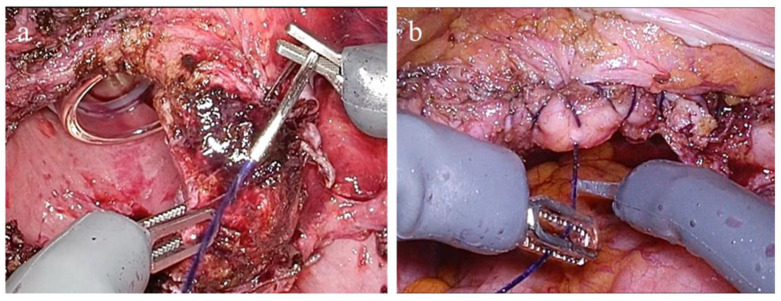
Intraoperative images during vaginal cuff suturing using the Saroa surgical system. (**a**) Suturing in progress at the vaginal cuff. (**b**) Completion of vaginal cuff closure.

This configuration enabled precise instrument manipulation and intuitive control of the grasping force throughout the procedure. Representative intraoperative views of vaginal cuff suturing are displayed in Figure 4. During this step, the surgeon perceives the grasping force applied to the vaginal cuff in real time through the system’s haptic feedback functionality. This sensory input allows for delicate tissue handling without excessive tension or the risk of suture breakage. The ability to modulate the grasping force throughout the suturing process enables secure and precise closure, contributing to atraumatic and controlled management of the vaginal cuff.

A vessel-sealing system was employed through the auxiliary port for vascular processing in all cases. In cases 4 and 5, the bipolar forceps of the Saroa surgical system became clinically available and were utilized to assist with hemostasis. In contrast, in cases 1–3, hemostasis was achieved solely with a vessel-sealing device operated by the assistant. The surgical approach employed in this study resembled a hybrid robot-assisted surgery model, integrating both robotic and conventional laparoscopic techniques. In this configuration, while the primary procedural steps were executed using the Saroa surgical system, certain tasks, such as the transection of major vessels, were performed by the assistant through conventional laparoscopic access ports. A dedicated vessel-sealing system for the Saroa surgical platform is currently under development.

### 2.4. Data Collection and Statistical Analysis

Demographic variables, operative duration (setup, console, and total), estimated blood loss, complications, and length of hospital stay were recorded. Inferential statistical analysis was not performed due to the limited sample size.

## 3. Results

### 3.1. Patient Characteristics

The cohort included five women aged 48–55 years, with a median age of 49 years. The patient background characteristics are summarized in Table 1. The diagnoses included three uterine myomas, one endometrial hyperplasia, and one endometrial polyp. Two patients had undergone prior abdominal or laparoscopic surgery. The median body mass index of the five patients was 23.9 kg/m^2^, ranging from 17.4 to 25.8 kg/m^2^. This indicates that the cohort comprised individuals ranging from underweight to mildly overweight based on the World Health Organization criteria. No major comorbidities were noted.

### 3.2. Surgical Outcomes

**Figure 5:**
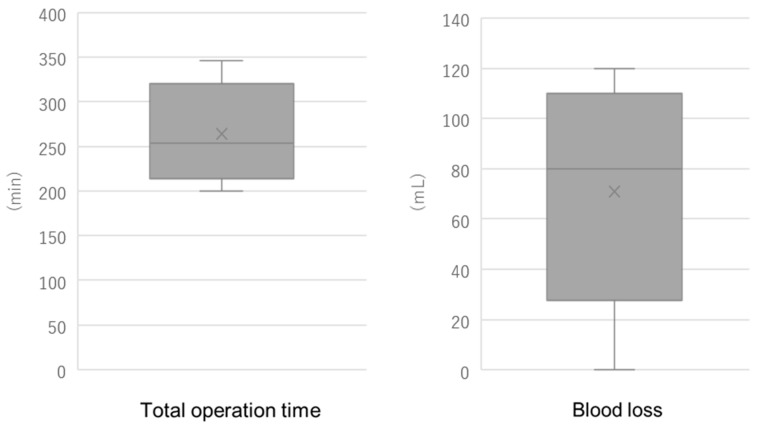
Distribution of total operative time and estimated blood loss in five robot-assisted hysterectomy cases. Box plots represent the interquartile range (IQR), with the horizontal line indicating the median and the cross symbol (×) representing the mean. Whiskers indicate the minimum and maximum values.

Table 2 summarizes the operative parameters for the five cases, including setup time, console time, total operative time, and estimated intraoperative blood loss. Setup time was defined as the interval from skin incision to robotic docking and instrument preparation. The console time was defined as the period during which the surgeon performed the operative maneuvers using the robotic console. Total operative time included the entire duration from skin incision to wound closure. The median setup time was 12 min, the median console time was 206 min, and the median total operative time was 254 min. The interquartile range (IQR) for total operative time was 227–294 min, with an overall range of 200 to 346 min, indicating moderate variability among cases. The IQR for console time was 143–211 min, also indicating moderate variability, which we attribute to the learning process during the initial clinical adoption of the Saroa system (Figure 5). The median estimated blood loss was 80 mL, with an IQR of 55–100 mL and a range of 0–120 mL, suggesting a relatively low and consistent intraoperative bleeding profile. No intra- or postoperative complications were observed. All patients were discharged on postoperative day four in accordance with the established clinical pathway. Long-term follow-up assessments at both one month and six months postoperatively revealed no complications or adverse events, and all patients reported favorable recovery without functional impairments or persistent symptoms.

### 3.3. Surgeon Feedback

Surgeons reported enhanced precision in tissue handling, particularly during grasping and manipulation of the vaginal cuff. The ability to modulate force in real time enables delicate handling without excessive traction or evidence of traction-related tissue injury. Mild fatigue in the fingers and forearms due to pneumatic resistance was observed during prolonged procedures with high feedback gain. However, the system allowed intraoperative adjustment of the feedback intensity to accommodate the surgeon’s preference. In contrast to conventional robotic surgery, where haptic feedback modulation is unavailable, the ability to tailor haptic feedback intraoperatively is subjectively associated with reduced physical strain. Although objective force measurements were not recorded in this initial case series owing to the absence of ethical approval for data acquisition, grasping force could be monitored intraoperatively, and intraoperative outcomes such as minimal blood loss and the absence of vascular or visceral injury suggested appropriate and nuanced force application. The system is capable of recording the grasping force in Newtons (Figure 3b), a feature that holds promise for future applications in surgical training, skill assessment, and feedback-based performance evaluation.

## 4. Discussion

This study demonstrated that the Saroa surgical system is safe and feasible for RAH. Although we previously performed robotic surgery using the da Vinci Surgical System (Intuitive Surgical, Inc., Sunnyvale, CA, USA), the initial clinical outcomes achieved with the Saroa surgical system were comparable to those observed during the early adoption of da Vinci-assisted procedures.

The Saroa surgical system is characterized by two principal advantages.

First, the system incorporated real-time haptic feedback. In this system, a pneumatic cylinder mounted on a robotic arm actuated the wires embedded within the instrument shaft and transmitted force to the grasping jaw at the instrument tip [3]. The grasping force was generated by the pressure inside the pneumatic cylinder, which was continuously monitored and converted into an electrical signal. These signals were then transmitted to the surgeon’s hand controller, where they were used to reproduce the forces applied at the instrument tip, thereby enabling real-time haptic feedback during surgical manipulation. The Saroa surgical system delivers real-time pneumatic grip force feedback, in contrast to the da Vinci 5 System, which primarily relies on software-mediated sensing to estimate contact and traction forces. This allows surgeons to perceive and modulate the pressure applied during tissue handling physically, thereby offering a direct and intuitive tactile experience [4]. A preclinical study by Sakai et al. demonstrated that the Saroa surgical system’s real-time contact-force feedback significantly reduced excessive tissue compression and enhanced precision in grasping maneuvers compared with conventional robotic systems lacking haptic feedback [5]. Histological assessments confirmed that such feedback substantially decreased tissue trauma, providing experimental evidence for the system’s potential to mitigate injury. These results complement the qualitative impressions reported in the present clinical series. In addition to minimizing tissue injury, the ability of the system to assess the grasping force in Newtons provides a quantifiable parameter for evaluating intraoperative tissue handling. This creates opportunities to investigate the relationship between the applied force and surgical proficiency. Notably, novice surgeons appear to benefit most from real-time perception of the grasping force, which may aid in preventing inadvertent tissue overcompression [6]. Such feedback facilitates precise tissue handling and may accelerate skill acquisition. For surgeons transitioning from systems without haptic feedback, a brief adaptation period is anticipated; however, the intuitive nature and real-time presentation of the feedback suggest a relatively shallow learning curve. This feature has the potential to enhance training for inexperienced surgeons and refine precision in experienced operators. These findings suggest that the Saroa surgical system could serve as a particularly valuable platform for surgical education and skill acquisition, offering both safety advantages and objective feedback for performance evaluation. Although the system can record the grasping force in Newtons, data collection was not performed in this initial case series due to the regulatory and ethical constraints specific to our institution. Future studies with prospective approval are planned to validate the force-based metrics. In Japan, this system has been introduced not only for gynecologic surgery but also for gastrointestinal, thoracic, and urologic procedures, all of which have been performed safely [7,8,9]. Unlike procedures in other specialties, gynecologic surgery frequently involves manipulation of the uterus, a relatively firm and heavy organ, and requires delicate maneuvers including grasping and suturing the vaginal cuff. Haptic feedback may be particularly advantageous during vaginal cuff suturing to avoid application of excessive force. These characteristics can benefit from real-time grasping force feedback, potentially improving surgical control and reducing the risk of tissue trauma [10].

Second, the Saroa surgical system features a compact and lightweight design, offering advantages in terms of cost efficiency and maneuverability, making the system particularly well suited for patients with small stature and low body weight, including slender adults. In contrast to traditional robotic systems that require docking, the Saroa surgical system is equipped with a lightweight patient cart that can be easily positioned between the patient’s legs. Weighing approximately 500 kg, the patient cart allows simple repositioning at the bedside without the need for complex docking procedures, thereby improving the operative efficiency [11]. The compact design further improves maneuverability, particularly in operating rooms with limited space. Moreover, the system proves to be cost-effective and adaptable, rendering it a promising option for expanding access to RAS, particularly where conventional robotic systems are less readily available.

However, despite these benefits, a potential limitation of the Saroa surgical system includes the use of only three robotic arms, compared to the four arms available on conventional platforms such as the da Vinci Xi. In complex procedures, the absence of a fourth arm could theoretically limit the ability to perform retraction and dissection simultaneously, potentially requiring additional assistant maneuvers. However, in our experience with RAH, the compact three-arm design did not create significant challenges in retraction, as its smaller footprint reduced interference with assistant instruments and allowed precise and timely traction according to the surgeon’s instructions. Nevertheless, in more advanced procedures involving multiple dissection planes or extensive adhesiolysis, strategic operative planning may be necessary to mitigate this structural limitation. An additional single-arm auxiliary unit is currently under development to complement the existing three-arm system, with the aim of enhancing surgical versatility and overcoming current limitations in tissue retraction. The Saroa surgical system presents an accessible and pragmatic solution for broad clinical implementations. Future comparative studies are warranted to delineate the specific procedural advantages and cost–benefit profiles of each robotic system, including the potential utility of force-based metrics for surgical evaluation and training.

### 4.1. Clinical Implications and Future Directions

The integration of real-time force measurements into clinical robotics represents a major step toward quantitative data-driven surgery. In the future, intraoperatively recorded grasping force profiles may enable the creation of standardized metrics for evaluating surgical performance across institutions. Furthermore, these metrics may facilitate personalized training protocols and early detection of suboptimal techniques. Compared to existing robotic surgical systems without haptic feedback, platforms such as the Saroa system offer potential advantages in improving tissue handling precision and enhancing surgical safety. Continued investigation involving large cohorts and diverse procedures is needed to validate the clinical impact of these capabilities and to clarify their comparative benefits over conventional systems, as well as to define the optimal ranges of grasping force for varying tissue types.

In the context of other next-generation robotic platforms, our median operative time and blood loss fall within the ranges reported during early clinical adoption. For hysterectomy performed with the Hugo™ RAS system (Medtronic, Inc., Minneapolis, MN, USA), a median operative time of 127 min (range: 98–255 min) and a median estimated blood loss of 50 mL (range: 30–125 mL) have been reported [12]. For the Versius system (CMR Surgical Ltd., Cambridge, UK), the median skin-to-skin operative time was 158.5 min (range: 50–385 min). When stratified by case sequence, the median operative time decreased from 191.5 min (range: 67–280 min) in the first 20 cases to 147 min (range: 90–273 min) in the last 20 cases, demonstrating a progressive reduction along the learning curve [13]. For the Senhance system (Asensus Surgical, Inc., Research Triangle Park, NC, USA), the total operative time for hysterectomy cases ranged from 32 to 136 min, with a median of 57 min. The estimated blood loss ranged from 25 to 100 mL, with a median of 75 mL [14]. Moreover, multi-platform comparisons suggest that newer systems (e.g., Hugo™, hinotori™, da Vinci SP™) yield broadly comparable perioperative outcomes in early use, although operative time may differ among systems and centers [15]. Taken together, these data indicate that our early experience with Saroa, which showed a median blood loss under 100 mL and acceptable variability in operative time, is consistent with international reports from the introductory phases of other novel robotic systems.

### 4.2. Strengths and Limitations

The first clinical application of the Saroa surgical system in the RAH was evaluated using haptic feedback. This was a single-institution study with short-term follow-up. One important limitation of this study is the reliance on subjective feedback from the operating surgeons to assess the utility of the haptic feedback system. While qualitative impressions offer valuable insights, they are inherently influenced by surgeon experience, expectations, and potential confirmation bias, especially in early-phase evaluations. Without objective data—such as force measurements or validated performance metrics—the reported improvements cannot be quantitatively confirmed. Future studies using blinded assessments and standardized tools will be necessary to reduce bias and more accurately evaluate the system’s impact.

## 5. Conclusions

This first clinical report on the Saroa surgical system highlights the safety, usability, and unique features of the system, particularly haptic feedback. Future studies with large sample sizes are warranted to systematically collect haptic feedback data and evaluate the system’s potential contributions to tissue-sparing techniques and patient-centered surgical outcomes. Future studies are planned to include prospective trials with intraoperative force data collection, enabling objective evaluation of tissue handling and further validation of the clinical benefits of haptic feedback.

## Figures and Tables

**Table 1:** The demographic and preoperative characteristics of the patients who underwent robot-assisted laparoscopic hysterectomy using the Saroa surgical system.

Case	Age (Years)	Diagnosis	Height (cm)	Weight (kg)	BMI (kg/m^2^)
1	49	Uterine myoma	162.2	65.2	24.8
2	49	Endometrial hyperplasia	164.7	47.3	17.4
3	48	Uterine myoma	158.5	64.7	25.8
4	54	Uterine myoma	158.6	60.2	23.9
5	55	Endometrial polyp	157.5	49.4	19.9

BMI, body mass index.

**Table 2:** Operative parameters and variability across cases.

Case	Setup Time (min)	Console Time (min)	Total Operative Time (min)	Blood Loss (mL)
1	10	211	294	80
2	12	143	227	100
3	12	276	346	120
4	15	206	254	55
5	29	133	200	0
Median (IQR)	12 (11–19)	206 (143–211)	254 (227–294)	80 (55–100)

min, minutes; mL, milliliters; IQR, interquartile range.

## Data Availability

The data that support the findings of this study are available from the corresponding author upon reasonable request.

## Abbreviations

The following abbreviations are used in this manuscript:

RAS	Robot-assisted surgery
RAH	Robot-assisted hysterectomy

## References

[1] Grasso J. (2025). Robot-assisted surgery: Past, present, and future. Digital Health.

[2] Iwai T., Kanno T., Miyazaki T., Haraguchi D., Kawashima K. (2020). Pneumatically driven surgical forceps displaying a magnified grasping torque. Int. J. Med. Robot..

[3] Ueda Y., Miyahara S., Tokuishi K., Nakajima H., Waseda R., Shiraishi T., Sato T. (2023). Impact of a pneumatic surgical robot with haptic feedback function on surgical manipulation. Sci. Rep..

[4] Intuitive Surgical Inc. da Vinci 5. https://www.intuitive.com/en-us/products-and-services/da-vinci/5.

[5] Sakai Y., Tokunaga M., Yamasaki Y., Kayasuga H., Nishihara T., Tadano K., Kawashima K., Haruki S., Kinugasa Y. (2024). Evaluating the benefit of contact-force feedback in robotic surgery using the Saroa surgical system: A preclinical study. Asian J. Endosc. Surg..

[6] Nakashima H., Ueda Y., Miyanari Y., Nishihara T., Hamasaki M., Ohbu M., Kawashima K., Yamakage H., Miyahara S., Tokuishi K. (2025). In vivo evaluation of tissue damage from varying grasping forces using the Saroa surgical system. Sci. Rep..

[7] Hanaoka M., Kinugasa Y., Sakai Y., Tokunaga M. (2023). World’s first report of sigmoidectomy for sigmoid cancer using the Saroa surgical system with tactile feedback. Update Surg..

[8] Ueda Y., Miyahara S., Tokuishi K., Nakajima H., Waseda R., Shiraishi T., Sato T. (2024). First clinical application of a surgical robot with haptic force feedback function for thoracic surgery: A case report. Shanghai Chest.

[9] Iwatani K., Urabe F., Saito S., Kawano S., Yamasaki T., Kimura S., Otsuki H., Fujio K., Kimura T., Miki J. (2024). Initial experience of a novel surgical assist robot “Saroa” featuring tactile feedback and a roll-clutch system in radical prostatectomy. Sci. Rep..

[10] Yamasaki Y., Tokunaga M., Sakai Y., Kayasuga H., Nishihara T., Tadano K., Kawashima K., Haruki S., Kinugasa Y. (2024). Effects of a force feedback function in a surgical robot on the suturing procedure. Surg. Endosc..

[11] Riverfield Inc. Saroa Surgical System. https://riverfieldinc.com/en/products/p04/.

[12] Monterossi G., Pedone Anchora L., Oliva R., Fagotti A., Fanfani F., Costantini B., Naldini A., Giannarelli D., Scambia G. (2023). The new surgical robot Hugo™ RAS for total hysterectomy: A pilot study. Facts Views Vis. Obgyn..

[13] Borse M., Godbole G., Kelkar D., Bahulikar M., Dinneen E., Slack M. (2022). Early evaluation of a next-generation surgical system in robot-assisted total laparoscopic hysterectomy: A prospective clinical cohort study. Acta Obstet. Gynecol. Scand..

[14] McCarus S.D. (2021). Senhance robotic platform system for gynecological surgery. JSLS.

[15] Matsuura M., Nagao S., Kurokawa S., Tamate M., Akimoto T., Saito T. (2024). Early outcomes of three new robotic surgical systems in patients undergoing hysterectomy. Updates Surg..

